# Varicella and Herpes Zoster in Madrid, based on the Sentinel General Practitioner Network: 1997–2004

**DOI:** 10.1186/1471-2334-7-59

**Published:** 2007-06-15

**Authors:** Napoleón Pérez-Farinós, María Ordobás, Cristina García-Fernández, Luis García-Comas, Soledad Cañellas, Inmaculada Rodero, Ángeles Gutiérrez-Rodríguez, Juan García-Gutiérrez, Rosa Ramírez

**Affiliations:** 1 Department of Epidemiology, Madrid Public Health Institute, Julián Camarillo 4B, 28037 Madrid, Spain

## Abstract

**Background:**

Varicella (chickenpox) is the primary disease caused by varicella-zoster virus. It is extremely contagious and is frequent in children. Indeed, in the absence of vaccination, a high proportion of the population is liable to contract it. Herpes zoster -more frequent among adults- is caused by reactivation of the latent virus. The objective of this study is to describe the status of and time trend for varicella and herpes zoster in the Madrid Autonomous Region prior to the introduction of the vaccine to the general population.

**Methods:**

Data source: individualised varicella and herpes zoster case records kept by the Madrid Autonomous Region Sentinel General Practitioner Network for the period 1997–2004. Cumulative incidences, crude and standardised incidence rates, and age-specific rates of varicella and herpes zoster were calculated for each year. Kendall's Tau-b correlation coefficient was calculated to evaluate whether incidence displayed a time trend. Spectral density in the time series of weekly incidences was estimated using a periodogram.

**Results:**

Standardised annual varicella incidence rates ranged from 742.5 (95% CI: 687.2 – 797.7) to 1239.6 (95% CI: 1164.5 – 1313.4) cases per 100 000 person-years. Most cases affected children, though complications were more frequent in adults. Varicella incidence displayed an annual periodicity but no trend over time. Most herpes zoster cases occurred at advanced ages, with incidence registering a rising annual trend but no seasonality factor.

**Conclusion:**

In the absence of vaccination, no significant changes in varicella incidence were in evidence recent years, though these were observed in the incidence of herpes zoster. Sentinel general practitioner networks are a valid instrument for surveillance of diseases such as varicella. Further varicella vaccination-coverage and vaccine-efficacy studies are called for.

## Background

Varicella (chickenpox) is the primary disease caused by the varicella-zoster virus. It mainly affects children and, in most cases, its clinical features include mild vesicular exanthema. Although less frequent among adults, the disease can nevertheless be more severe, leading to a greater number of complications which may even prove life-threatening[1]. Due to its extreme contagiousness, in the absence of vaccination most people have suffered from the disease by the time they reach adulthood.

Herpes zoster is a secondary infection caused by reactivation of the latent virus, and principally affects adults[1,2]. It is less frequent than varicella, and the risk of suffering herpes zoster over the course of a lifetime has been estimated between 20 at 30%[1,3].

The introduction of universal varicella vaccination in children has been controversial, owing to its possible effects on the disease itself and on herpes zoster[4]. Initially the vaccine was exclusively used on immunodepressed patients, but subsequently its general use was authorised in a number of countries, including Japan, South Korea (1988)[5], United States[6], Sweden and Germany (1995). In Spain it was first introduced in 1997 for high-risk patients, and in 2005 it was included in the vaccination schedule for administration to adolescents who had not suffered the illness, whereas the Spanish Paediatric Association as been recommending vaccination in children as from the age of 12 months since 1999[7]. Recently, the Madrid Autonomous Region (MAR) has included universal varicella vaccination in its systematic vaccination schedule for children aged 15 months[8]. According to a series of studies, the varicella vaccine now in use in various countries is effective[9,10]. As regards the immunity conferred, some studies indicate that it is high[5] but this is not yet clear[10] and it would seem that at least two doses of vaccine are needed[11,12].

A vaccine against herpes zoster and post-herpetic neuralgia is currently being developed, and it is not well known about how this may influence the possible effect of varicella immunisation on herpes zoster and, as a result, on the indications and effectiveness of the herpes zoster vaccine[13,14].

Sentinel networks are an increasingly useful tool in epidemiological surveillance and research. The MAR Sentinel General Practitioner Network (SGPN) came into operation in 1991, and the reporters are volunteer physicians.

This study sought to describe the status of and evolution for varicella and herpes zoster in the MAR, based on case reports of the SGPN, from 1997, the year in which these diseases first came under surveillance, until 2004, prior to the introduction of the varicella vaccine into the general population.

## Methods

The Madrid Region Sentinel General Practitioner Network is made up of general practitioners and primary care paediatricians who voluntarily submit weekly reports of cases of varicella and herpes zoster, as well as other diseases. The population attended by these physicians is representative, in terms of age and gender, and accounts for approximately 2% of the total population of the MAR.

### Case definitions

A case of varicella was defined as an acute disease with moderate fever (< 38.5°C) and vesicular exanthema, which evolves in the form of outbreaks, and gives rise to lesions that swiftly develop from superficial papules into vesicles, and occasionally into scabs. A case of herpes zoster was defined as a vesicular rash, generally unilateral, with dermatomeric distribution.

Apart from age and sex, data recorded in varicella cases included previous exposure to other cases of varicella or herpes zoster, place of exposure (if known), as well as complications suffered (bacterial infection, pneumonia, and neurological and other complications).

The following were calculated for both varicella and herpes zoster: cumulative weekly incidences for the overall study period; and, crude and standardised incidence rates for the standard European population and age-specific rates for each year.

Kendall's Tau-b correlation coefficient was calculated to evaluate whether annual varicella and herpes zoster incidence described a trend over time. Similarly, spectral density in the time series of weekly incidences was estimated, using a periodogram and Tukey-Hamming smoothing.

SPSS^® ^12.0 statistical package was used.

This study complies with all current international ethic norms.

## Results

In the period from 1997 to 2004, a total of 9856 cases of varicella and 1798 cases of herpes zoster were reported to the Madrid Region SGPN.

**Figure 1:**
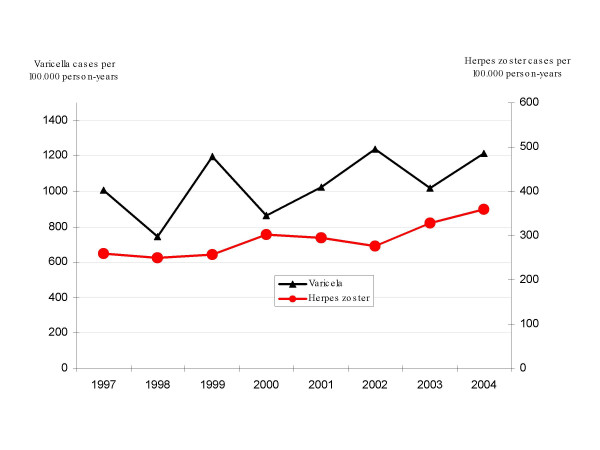
**Annual varicella and herpes zoster incidence in the Madrid Region. Madrid Sentinel General Practitioner Network**. Annual incidence rates of varicella and herpes zoster are presented. Rates are expressed in cases per 100,000 person-years. In the left axis are presented rates for varicella, and in the right axis, the rates for herpes zoster.

For varicella, the standardised annual incidence rates ranged from 742.5 (95% CI: 687.2 – 797.7) to 1239.6 (95% CI: 1164.5 – 1313.4) cases per 100000 person-years (Figure 1). Of this total, 51.4% of cases occurred among males and 48.6% among females.

**Figure 2:**
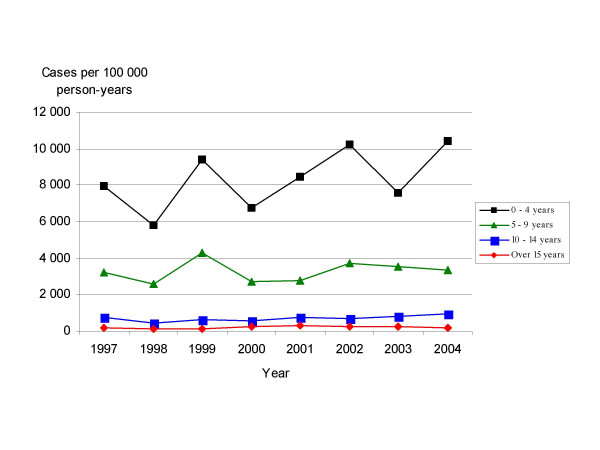
**Age-specific varicella incidence rates. Madrid Region Sentinel General Practitioner Network: 1997–2004**. Age-specific varicella incidence rates are expressed as cases per 100,000 person-years. The ages over 15 years have been joined as the incidence is very low.

Close on 60% of cases occurred among children under 5 years of age, and almost 90% among children under 10 years of age (Figure 2). 81% of cases with known exposure were exposed to another varicella case, 1.4% had been in contact with a herpes zoster case, and 17.7% belonged to outbreaks. The main places of exposure were nursery and school (74.3%), and home (25.0%).

**Figure 3:**
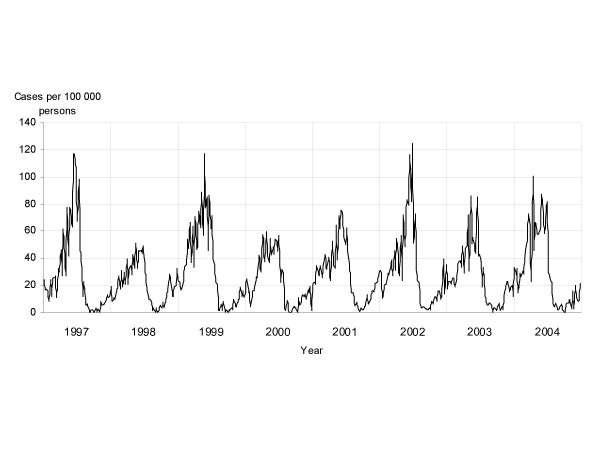
**Annual cumulative weekly incidences of varicella per 100,000 population. Madrid Region Sentinel General Practitioner Network: 1997–2004**. Weekly cumulative incidence has been expressed as cases per 100,000 persons.

**Figure 4:**
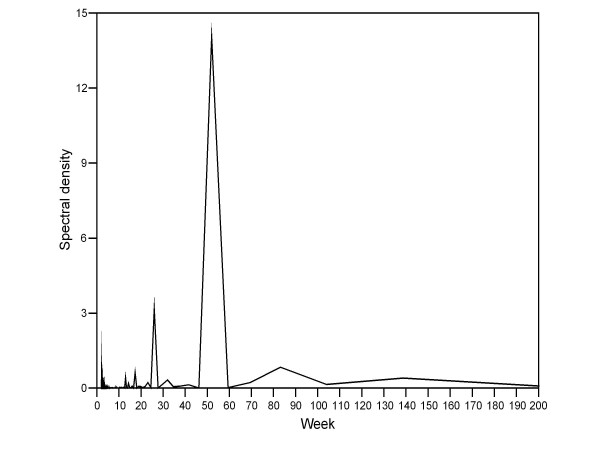
**Periodogram depicting the time series of cumulative varicella incidence. Madrid Region Sentinel General Practitioner Network: 1997–2004**. The figure shows the periodicity of peaks in varicella incidence using a week as time unit. The peaks in periodogram reveal when the pattern repeats.

Time-trend analysis yielded a Kendall Tau-b correlation coefficient for incidence and time of 0.43 (p = 0.138). The distribution of weekly varicella incidences depicted in Figure 3 suggests an annual periodicity. This same periodicity is observable in the varicella time series periodogram (Figure 4), which shows a high spectral density in the 52-week period.

Among the 9856 cases, 364 complications (3.7%) were reported. The highest percentages of complications were registered in respect of the oldest age groups (Table 1), and the greatest number consisted of cutaneous infections (68.6%).

**Table 1:** Varicella case complications detected by the Madrid Region Sentinel General Practitioner Network from 1997 to 2004, by age group.

	Age (years)							
	
	0–4	5–9	10–14	15–19	20–24	25–29	30–34	35 and over
Cutaneous overinfection	124	59	12	2	5	7	8	10
Pneumonia	17	5	1	1	1	0	2	0
Neurological complications	1	1	0	0	0	0	0	0
*Otitis media*	19	4	2	0	0	0	0	0
Conjunctivitis and other ophthalmological complications	14	9	3	0	0	0	0	0
Other	20	3	0	0	0	0	0	1
Total	195	81	18	3	6	7	10	11
Percentage of complicated cases of varicella	3.5	3.0	3.1	3.6	5.9	7.4	10.0	7.7

**Figure 5:**
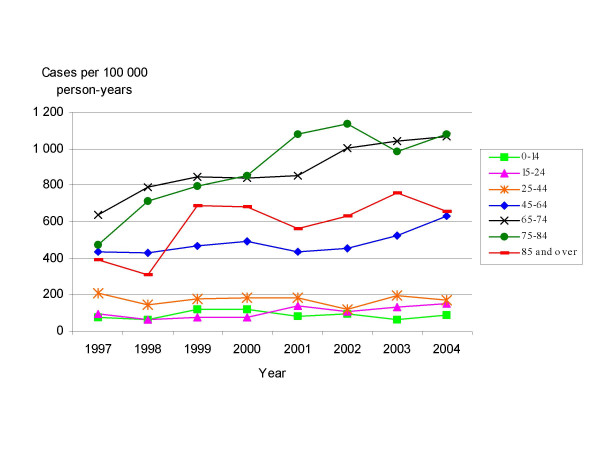
**Age-specific herpes zoster incidence rates. Madrid Region Sentinel General Practitioner Network: 1997–2004**. Age-specific herpes zoster incidence rates are expressed as cases per 100,000 person-years.

The standardised annual herpes zoster incidence rates ranged from 249.9 (95% CI: 217.9 – 282.0) to 359.4 (95% CI: 322.3 – 396.6) cases per 100000 person-years (Figure 1). In all, 59.2% of cases occurred among females, and 40.8% among males. The highest incidences were registered in respect of the oldest age groups (Figure 5). For the oldest age groups a rising trend was observed in both overall and specific incidences, a finding confirmed by a Kendall Tau-b correlation coefficient of 0.64 (p = 0.026). Unlike varicella, there was no evidence of seasonality in the herpes zoster time series.

For varicella, 0.2 % of cases carried no record of patient's gender, and 5.2% had missing values of age. For herpes zoster 0.1% of missing values were founded in patient's gender.

## Discussion

There was no significant shift in trend in annual incidence of varicella in Madrid from 1997 to 2004, despite the fact that the annual rates display rises and falls. As this pattern was almost identically reproduced in children aged under 5 years, among whom incidence is highest, the analysis was not repeated for that age group. An important seasonality factor was in evidence, with peaks in incidence from May to July, and lows towards October. Distribution by gender showed no differences. These results are similar to others obtained in other countries [15-17].

Few complications were observed, and these mostly consisted of bacterial skin overinfections. Note should however be taken of the fact that the most severe complications were least frequent. Hence, the SGPN may conceivably not detect all complications because it works with a representative sample that covers approximately 2% of the population of Madrid. Moreover, most of complications of varicella receive hospital attention and are not collected by the SGPN[18].

In contrast to varicella, herpes zoster registered a sustained and significant rise in annual incidence, particularly among the oldest age groups, though there was no evidence of seasonality[2]. Again unlike varicella, and as described elsewhere, there was a higher percentage of cases among females than males[2,19].

The universal vaccination is currently a widely debate subject, with objections[13,20,21] and defenders[22,23], including clinical and cost-effectiveness aspects [24-27].

Starting from data as presented in this work, further studies are called for to evaluate the vaccine, its effectiveness and the duration of immunity.

The number of Sentinel Networks has grown worldwide since the 1960's and they are used in fields as diverse as communicable [28-30] and non-communicable disease surveillance[31,32]. In Madrid, SGPN has demonstrated to be an efficient and useful system for the epidemiologic surveillance of several diseases, and especially for varicella and herpes zoster.

## Conclusion

In recent years, the time trend in respect of varicella incidence among subjects who were not covered by universal varicella vaccination has remained stable. Although the percentage of complications is not high, these are nevertheless more frequent among adults. Incidence of herpes zoster maintained a rising trend over the study period.

Sentinel general practitioner networks are useful instruments for epidemiological surveillance of diseases such as varicella and herpes zoster.

## Competing interests

The author(s) declare that they have no competing interests.

## Authors' contributions

NPF, MO, CGF, LGC and SC have made substantial contributions to study conception, statistical analysis, and interpretation of results, as well as in drafting and revising the manuscript. IR, AGR, JGG and RR have contributed especially to acquisition of data, maintenance of information systems and revision of the manuscript. All authors read and approved the final manuscript.

## Pre-publication history

The pre-publication history for this paper can be accessed here:


